# Are medical students more prone to symptoms of anxiety and depression?

**DOI:** 10.1192/j.eurpsy.2025.1023

**Published:** 2025-08-26

**Authors:** S. Kucukalic, M. Arnautovic Tahirovic, A. Ganibegovic, M. Bubenik, A. Tahirovic

**Affiliations:** 1 Psychiatric clinic, Clinical centre university of Sarajevo; 2 Psychiatric hospital of Canton Sarajevo; 3 Faculty of Medicine University of Sarajevo, Sarajevo, Bosnia and Herzegovina

## Abstract

**Introduction:**

Faculty of Medicine is often recognized as one of the most laborious. Student workload, constant exposure to stressful situations, fear of failure, pressure from parents, exposure to death and human suffering are some of the many factors associated with increasing levels of anxiety and depression among medical students.

**Objectives:**

The aim of this study was to estimate the severity of anxiety and depression disorders among medical students.

**Methods:**

In this cross-sectional study, self-report anonymous online survey was distributed to the students of all six years of the Faculty of Medicine, University of Sarajevo. In this survey, which consisted of 33 questions, we gathered general information and the Beck’s Anxiety and Depression Inventory were used to assess the severity of anxiety and depression, whereby respondents received points by answering each question, and after scoring, they were categorized in different groups based on the severity of anxiety and depression.

**Results:**

129 students completed the survey. Considering the Beck’s Anxiety Inventory, 56% scored for the “Low anxiety” group, 33% scored for the “Moderate Anxiety” group, 11% scored for the “Potentially concerning levels of anxiety”. Considering the Beck’s Depression Inventory, 43% of the students scored for the group “These ups and downs are considered normal”, 21% scored for “Mild mood disturbances”, 9% for the “Borderline clinical depression”, 19% for “Moderate depression”, 6% for “Severe depression”, 2% for “Extreme depression”.

**Conclusions:**

Psychiatric morbidity found needs to be identified and treated at the earliest, because it can lead to suicidal ideation. Medical students should be encouraged to seek help and adequate facilities should be available to all of them.

**Disclosure of Interest:**

None Declared

